# Evaluation of a New Survivin ELISA and UBC^®^ *Rapid* for the Detection of Bladder Cancer in Urine

**DOI:** 10.3390/ijms19010226

**Published:** 2018-01-11

**Authors:** Jan Gleichenhagen, Christian Arndt, Swaantje Casjens, Carmen Meinig, Holger Gerullis, Irina Raiko, Thomas Brüning, Thorsten Ecke, Georg Johnen

**Affiliations:** 1 Institute for Prevention and Occupational Medicine of the German Social Accident Insurance, Institute of the Ruhr-University Bochum (IPA), 44789 Bochum, Germany; casjens@ipa-dguv.de (S.C.); meinig@ipa-dguv.de (C.M.); raiko@ipa-dguv.de (I.R.); bruening@ipa-dguv.de (T.B.); johnen@ipa-dguv.de (G.J.); 2 Department of Urology, Lukaskrankenhaus Neuss, 41464 Neuss, Germany; CArndt@lukasneuss.de; 3 University Hospital for Urology, Klinikum Oldenburg, 26133 Oldenburg, Germany; holger.gerullis@gmx.net; 4 Department of Urology, HELIOS Hospital, 15526 Bad Saarow, Germany; thorsten.ecke@helios-gesundheit.de

**Keywords:** survivin, UBC^®^*Rapid*, bladder cancer, urine, biomarker combination, non-invasive

## Abstract

Urine-based biomarkers for non-invasive diagnosis of bladder cancer are urgently needed. No single marker with sufficient sensitivity and specificity has been described so far. Thus, a combination of markers appears to be a promising approach. The aim of this case-control study was to evaluate the performance of an in-house developed enzyme-linked immunosorbent assay (ELISA) for survivin, the UBC^®^ *Rapid* test, and the combination of both assays. A total of 290 patients were recruited. Due to prior bladder cancer, 46 patients were excluded. Urine samples were available from 111 patients with bladder cancer and 133 clinical controls without urologic diseases. Antibodies generated from recombinant survivin were utilized to develop a sandwich ELISA. The ELISA and the UBC^®^ *Rapid* test were applied to all urine samples. Receiver operating characteristic (ROC) analysis was used to evaluate marker performance. The survivin ELISA exhibited a sensitivity of 35% with a specificity of 98%. The UBC^®^ *Rapid* test showed a sensitivity of 56% and a specificity of 96%. Combination of both assays increased the sensitivity to 66% with a specificity of 95%. For high-grade tumors, the combination showed a sensitivity of 82% and a specificity of 95%. The new survivin ELISA and the UBC^®^ *Rapid* test are both able to detect bladder cancer, especially high-grade tumors. However, the performance of each individual marker is moderate and efforts to improve the survivin assay should be pursued. A combination of both assays confirmed the benefit of using marker panels. The results need further testing in a prospective study and with a high-risk population.

## 1. Introduction

Bladder cancer is the most common cancer of the urinary tract and ranks fifth among cancers in men in western countries [1]. It is typically diagnosed by cystoscopy followed by pathological examination of suspicious tissue. Cystoscopy is a time-consuming method that requires an experienced urologist to perform and can be painful for the patients. Thus, there is a need for an easier and non-invasive diagnostic method. Being minimally- or non-invasive is a key characteristic of biomarkers, facilitating the use of easily accessible body fluids instead of tissue, which requires invasive sampling. Because of its close proximity to the target organ, it is of general acceptance that urine might be a good source for bladder cancer-specific biomarkers [2].

Based on urine or urinary cells, only a few molecular markers have been approved by the federal Food and Drug Administration (FDA) so far. In addition, these markers are designated merely as supporting tools for monitoring bladder cancer patients instead of replacing cystoscopy [3,4]. The nuclear mitotic apparatus protein 1 (NUMA1) can be quantitated by the commercial NMP22^®^ test or detected by the BladderChek^®^ test [5]. The reported sensitivities of the NMP22 assays in case-control studies vary between 47% and 100%, while the specificities range from 58% to 91% [6,7]. In contrast to the results from case-control studies, in a prospective study (UroScreen), the assay reached a sensitivity of 97% and a specificity of 29% in prediagnostic samples [7]. The performance of NMP22 was limited by a relative high number of false-positive test results, which can be caused, for example, by hematuria or infections [5,7]. Using fluorescence in situ hybridization (FISH), the UroVysion test is applied to detect chromosomal copy number variations in exfoliated urothelial cells sedimented from urine samples. In a pooled analysis of FISH-based assays, the average sensitivity was 76% (65–84%) and the specificity 85% (78–92%) [8], another pooled analysis yielded 72% and 83%, respectively [9]. In the prospective study UroScreen, the UroVysion test reached a sensitivity of 45% at a specificity of 97% [10]. While UroVysion is relatively specific, it only showed a moderately increased sensitivity compared to the much cheaper classical cytology. It is also restricted by the requirement of special equipment and a time-consuming, expert-dependent procedure [11,12].

The limited performance of individual markers can be improved by combining two or more markers in a panel. For example, in the prospective study UroScreen, a combination of UroVysion and NMP22 reached a sensitivity of 67% and a specificity of 95% [11]. However, there is still a lack of easy to apply and cost-effective assays with adequate performance that would qualify as candidates for a biomarker panel.

In order to evaluate additional assays of urinary biomarkers, we focused on two candidates, UBC^®^ *Rapid* and survivin, and tested their ability to detect bladder cancer. UBC^®^ *Rapid* is a commercially available point-of-care test measuring cytokeratin fragments 8 and 18 in urine. Affordability and ease of use are factors that would support its implementation in routine diagnostics. Survivin is a well-known protein biomarker and detectable in almost every cancer [13,14], including bladder cancer [15,16]. Detailed information about biological function is discussed in several reviews [13,17] as well as its potential as a therapeutic target [18,19,20]. Most studies analyzing the expression of survivin used invasively obtained tissue samples or rely on the detection of survivin mRNA in sedimented cells from urine [21,22,23]. Because of our previous experience with the practical problems associated with the limited stability of mRNA, we decided to focus on the more stable survivin protein. A well-established method for the measurement of proteins in laboratory diagnostics is the enzyme-linked immunosorbent assay (ELISA). ELISAs for the detection of survivin are commercially available, but often lack sufficient performance, particularly when using urine as a sample matrix [24]. 

The aim of this study was to develop a new ELISA for the quantification of survivin in urine samples and to evaluate the performance of the ELISA in combination with UBC^®^ *Rapid* for urine-based detection of bladder cancer, especially high-grade tumors.

## 2. Results

### 2.1. Survivin Production and ELISA Development

**Figure 1:**
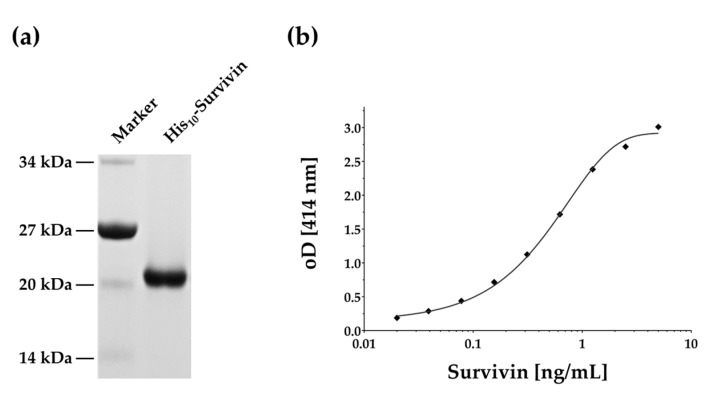
SDS-PAGE and ELISA reference curve. (**a**) Coomassie-blue stained SDS-PAGE of recombinant purified His_10_-survivin; (**b**) representative four-parameter logistic curve fit for the survivin ELISA.

Cloning, expression, and affinity purification yielded about 2.5 mg of His_10_-survivin fusion protein suitable for antibody generation in rabbits (Figure 1a). 

Antibodies were purified and partially biotinylated for application in a sandwich ELISA. No cross-reactivity was observed for anti-survivin antibodies. The best signal-to-noise ratio was obtained with a dilution of 1:5000 for the capture antibody and a dilution of 1:10,000 for the detection antibody. The resulting sigmoidal curve could be interpolated within the range of 0.025–5 ng/mL, with a limit of detection (LoD) of about 0.033 ng/mL for the new survivin ELISA (Figure 1b). We compared our ELISA with the commercially available survivin ELISA kits of R&D and Enzo Life Sciences by performing four-parameter logistic curve-fits for the reference curves. The standard curve of our ELISA was comparable to those of the commercial ELISA kits (Figure S1).

Reproducibility of the new survivin ELISA was investigated by measuring three defined samples of low, medium, and high survivin concentrations multiple times (*n* = 24). The resulting coefficients of variation (CVs) were 7.2% for the low, 4.6% for the medium, and 6.5% for the high concentration sample. In addition, we tested the repeatability with two different samples of medium survivin concentration at different time points. The resulting CVs were 8.8% and 19.4%. Additionally, matrix effects were investigated by spike-in experiments. Defined recombinant survivin concentrations were spiked into survivin-free urine samples. The recovery rate ranged from 74.8 to 88.9%. 

### 2.2. Study Group

Table 1 depicts the characteristics of the 244 participants, divided into tumor cases (*n* = 111), and clinical controls (*n* = 133). Most of the participants were men (*n* = 175). The median age of the tumor group and clinical controls was similar (74 vs. 71 years). 

Among all groups, median volume of urine was 30 mL with a retention time of about 2 h within the bladder before voiding. The median pH of urinary samples was 5.0 in all groups. Additional details regarding the tumor group are listed in Table 2. 

### 2.3. Survivin in Urine of Bladder Cancer Patients

Initially, we tested voided urine, supernatant, and urinary pellet for measurable amounts of survivin. Only urinary pellet exhibited detectable amounts of survivin. Therefore, all herein presented survivin-related data refer to the urinary pellet. 

**Figure 2:**
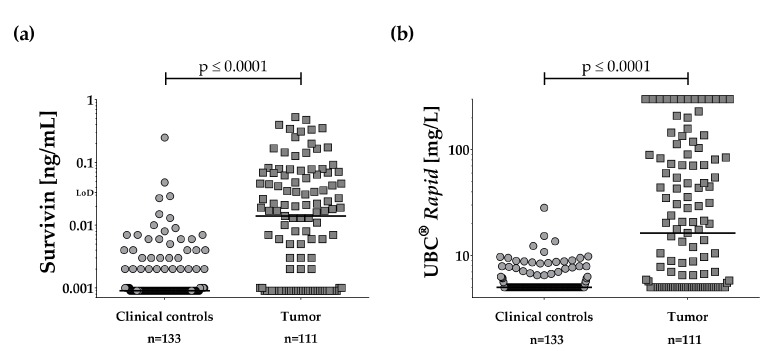
Dot plots of marker results in urine. Comparison of marker concentrations in urinary samples from clinical controls and bladder tumor patients. (**a**) Survivin and (**b**) UBC^®^ *Rapid*. Lines depict the median for each group. *p*-Values were obtained from the Wilcoxon signed-rank test.

The distributions of survivin concentrations in tumor and control groups are shown in Figure 2a. 

**Figure 3:**
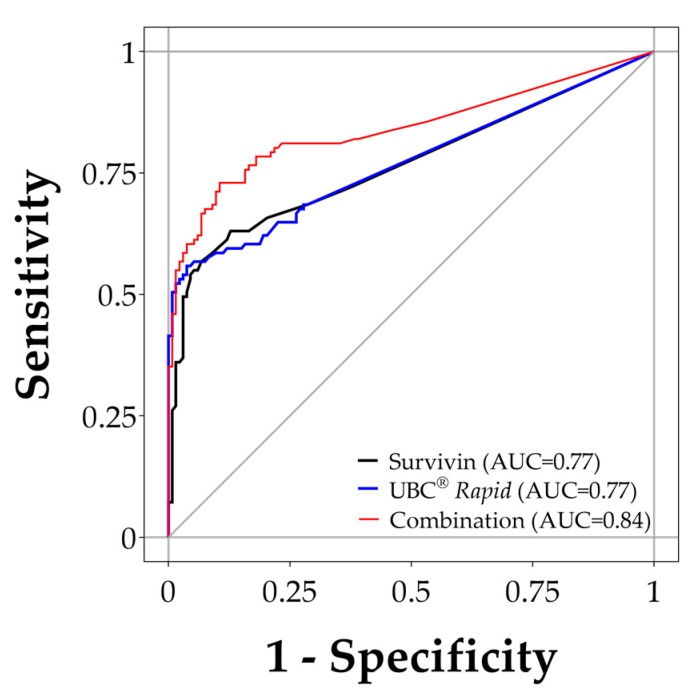
ROC analyses of survivin and UBC^®^ *Rapid*. ROC curves for survivin (AUC = 0.77), UBC^®^ *Rapid* (AUC = 0.77), and the combination of both assays (AUC = 0.84) based on comparing tumor and clinical control group.

The median survivin concentration was below the LoD (<0.033 ng/mL) in both groups. Normalization by creatinine or amount of total protein was therefore not attempted. However, the median survivin concentration differed between tumor group and clinical controls (*p* < 0.0001). Survivin levels were not influenced by obtained volume of urine, retention time within the bladder, or gender. Using a cut-off of 0.033 ng/mL, our survivin ELISA showed a sensitivity of 35% (39/111 tumors true-positive) and a specificity of 98%, when comparing tumor group and clinical controls. Tumor grade had an influence on the marker performance. The survivin ELISA detected 51% (28/55) of the high-grade tumors but only 18% (10/55) of the low-grade tumors (Table 3, Figure S2a). Grading data was missing for one case. The area under the curve (AUC) was 0.77 for the ROC curve analysis of tumor versus clinical controls (Figure 3).

### 2.4. Performance of UBC^®^ Rapid 

The distribution of the UBC^®^ *Rapid* results for the clinical controls and the tumor group are depicted as dot plots in Figure 2b. The median UBC^®^ *Rapid* value was <5.0 mg/L for clinical controls and 16.3 mg/L for the tumor group. The difference between the tumor group and the control group was statistically significant (*p* < 0.0001). Using the cut-off (10 mg/L) recommended by the supplier, the UBC^®^ *Rapid* assay reached a sensitivity of 56% (62/111) at a specificity of 96%. As seen with survivin, the sensitivity of the assay increased (to 73%) in high-grade tumors in comparison to controls (Table 3, Figure S2b). ROC curves and AUC values for the UBC^®^ *Rapid* test were similar to those of the survivin assay and are shown in Figure 3. 

### 2.5. Combination of Survivin and UBC^®^ Rapid

**Figure 4:**
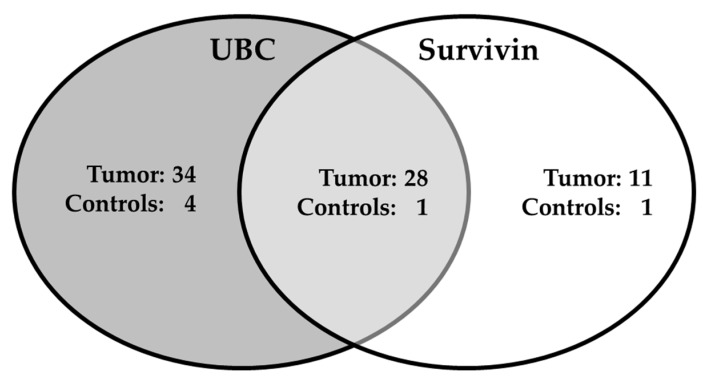
Venn diagrams of all positive test results. The cut-off for UBC^®^ *Rapid* was >10 mg/L (**left**) and for survivin >0.033 ng/mL (**right**).

Because of the generally limited performance of individual biomarker assays, we also looked at the possible benefits of a combination of biomarkers. To our knowledge, this is the first approach exploring survivin and UBC^®^ *Rapid* as a panel. The results of the combination of the survivin ELISA and the UBC^®^ *Rapid* test are shown in Table 3 and depicted in Figure 3. A scatter plot of the values of the tumor samples indicates a moderate correlation between both assays (Spearman’s r_s_ = 0.38; Figure S3). Further analysis by Venn-diagrams (Figure 4) revealed that, in addition to 28 tumors detected by both assays, 45 tumors were solely detected by either UBC^®^ *Rapid* (34 tumors) or survivin (11 tumors). From the ROC analysis, an AUC of 0.84, a sensitivity of 66% and a specificity of 95% could be derived for the combination. The difference between each single marker AUC and AUC of the combination is statistically significant (combination vs. survivin: *p* = 0.0025; combination vs. UBC^®^ *Rapid*: *p* = 0.0005). Comparing high-grade tumors with controls revealed an AUC of 0.91, a sensitivity of 82%, and a specificity of 95% (Table 3, Figures S4 and S5). Here, the difference between single marker AUC and combination AUC was not significant (combination vs. survivin: *p* = 0.1826; combination vs. UBC^®^ *Rapid*: *p* = 0.0685).

### 2.6. Possible Influence of Microhematuria

Microhematuria could be a possible influencing factor of biomarker performance. We therefore investigated the effect of microhematuria in the control group. Of the 133 controls, 28 had erythrocytes (between 5 and 250 cells [25]) in their urine. Median marker concentrations in controls with and without microhematuria did not show significant differences for both markers (*p* = 0.4511 for survivin and *p* = 0.4121 for UBC^®^ *Rapid*).

## 3. Discussion

Bladder cancer is one of the most common cancers worldwide [26]. The current gold standard for bladder cancer detection is cystoscopy and cytology. Those methods are routinely applied in patients with hematuria or other symptoms suggestive of bladder cancer [8]. Clinical symptoms are more likely to occur at later stages of tumor development. Biomarkers have the potential to detect cancer at earlier stages, facilitating an earlier and therefore more curative therapy that ideally results in decreased mortality or chance of recurrence, while potentially reducing the number of invasive diagnostic procedures [27]. Urine is apparently an ideal source for non-invasive biomarkers for detection or monitoring urothelial bladder cancer [2]. Screening for bladder cancer is medically reasonable for high-risk groups, e.g., persons with a high recurrence rate or a previous exposure to bladder cancer carcinogens [4,11,15,28]. For instance, in Germany, workers with a previous occupational exposure to aromatic amines are offered free medical exams to screen for bladder cancer [11].

Examples for urine-based biomarkers that have been suggested for screening are UroVysion, uCyt+, or NMP22. However, the markers had some drawbacks, including observer bias, time-consuming procedures, confounders, and/or large variations in reported performance [5,11,29,30]. Survivin is another possible target for biomarker development that has been well published [21]. Unfortunately, most studies relied on immunohistochemistry of tissue samples or mRNA-based approaches [21,22,23,30]. ELISA-based techniques are an easy-to-use assay format frequently used in routine applications for protein quantification, but less commonly applied to urinary samples [24]. To overcome the limitations associated with survivin mRNA [22], we investigated a newly developed ELISA to detect survivin at the protein level. The ELISA featured a LoD (0.033 ng/mL) and detection range comparable to commercially available ELISAs. We performed a four-parameter curve fit for the calibration curve because this procedure is commonly recommended and appears to be more reliable for values close to the LoD [31]. 

We could not observe detectable amounts of survivin in urinary supernatant or unprocessed voided urine using the new ELISA. We did not attempt to detect survivin in concentrated urine [32]. Instead, we based our survivin determination on measurements in sedimented cells from urine to avoid matrix effects that might influence the assay. Urine is a dynamic and heterogenic fluid comprising varying salt concentrations and pH values [33,34,35,36]. Those parameters are important factors for antibody–antigen interaction [37]. A secretion of survivin from tumor cells via exosomes has been reported recently [38]. An enrichment of exosomes from urine for the subsequent determination of survivin is an interesting approach that should be attempted in the future but may require a more sensitive assay. Currently, we are investigating an improved version of our assay based on a solid-phase proximity ligation assay. This technique replaces the enzymatic reaction of an ELISA with quantitative PCR by transferring the original protein signal to an amplifiable DNA signal [39,40].

The presented case-control study revealed a sensitivity of 35%, a specificity of 98%, and an AUC of 0.77 for our survivin ELISA. The results are in good agreement with the reported sensitivity and specificity by Ohsawa et al. [41]. In comparison, Li et al., Eissa et al., and Srivastava et al. reported a somewhat higher performance with AUC values of 0.85, 0.87, and 0.88, respectively [32,42,43]. The composition of the study populations—e.g., variations in grade and stage of the tumors and variations in the control group—might have contributed to these differences. As seen before with immunohistochemistry and mRNA-based assays, the survivin ELISA was more sensitive for high-grade tumors [22,23,30]. This is also plausible in view of the mechanistic role of survivin, which is involved in suppression of apoptosis as well as regulation of mitosis [13,16,17,44,45].

Parallel to survivin, we examined the commercial point-of-care test UBC^®^ *Rapid*. Compared to other commercially available tests like UroVysion or uCyt+, it is affordable, simple to use and not observer-biased. In our study group, the UBC^®^ *Rapid* test reached an AUC of 0.77 and a sensitivity of 56% at a specificity of 96%, using the recommended cut-off of 10 mg/L. This is in accordance with other reports showing sensitivities in the range of 53–64% and specificities in the range of 63–91% [46,47,48,49,50,51]. Similar to survivin, UBC^®^ *Rapid* also has a higher sensitivity for high-grade tumors [46,50,51,52] in comparison to low-grade tumors, indicating that the UBC^®^ *Rapid* test may be more useful for this subgroup. The test may be influenced by urinary tract infections as has been shown before [53].

Screening, even in high-risk populations, requires a high specificity in order to avoid unnecessary invasive diagnostic procedures and psychological burden for the patients, which can be a consequence of false-positive test results [52,54]. As has been reported before, no single marker has reproducibly shown a sufficiently high sensitivity in conjunction with the required high specificity to detect bladder cancer early [2,11]. This was also true for the two biomarkers we investigated in the present study. Both survivin and UBC^®^ *Rapid* had relatively good specificities but moderate sensitivities at the given cut-offs. However, lower sensitivities can be compensated by the use of several biomarkers in a panel. Indeed, the combination of both markers increased the sensitivity to 66% at a specificity of 95%. For the subgroup of high-grade tumors, the sensitivity even increased to 82%. Due to the fact that high-grade tumors have a higher rate of progression and/or recurrence, it is helpful to focus on markers exhibiting a higher sensitivity for this particular subgroup.

A limitation of the markers that has been reported before is the reduced performance associated with other urologic diseases, confounders that would also be expected in a typical clinical setting [45,50,55,56]. Microhematuria apparently does not affect the survivin ELISA or UBC^®^ *Rapid*. Another possible confounder for biomarker assays can be a urinary infection. A typical example is the protein marker NMP22 [5,7]. Because expression of survivin has been reported in some cases of inflammation [56] and links between inflammation pathways and cancer development are generally known [56,57], bladder infections could also influence urinary survivin concentrations. Therefore, a combination with other markers like UroVysion, which are less affected by confounders, and different algorithms to combine the markers may be interesting options. In addition, the aforementioned efforts to refine the survivin assay could lead to a higher sensitivity. An advantage of the survivin ELISA and UBC^®^ *Rapid* is their affordability and ease of use, factors that are important for a future application in screening or surveillance programs. The case-control design and the selection of the control group are limitations of our study, which was intended as an initial assessment of a new assay. Thus, before an application in screening is possible, the markers have to be validated in a prospective study with an independent and larger study population. The establishment of a suitable cohort is currently under way, but the process requires time and some effort.

## 4. Materials and Methods 

### 4.1. Study Population

Between January 2014 and July 2015, a total of 290 patients were recruited at the Lukaskrankenhaus (Neuss, Germany). Because of a prior bladder cancer diagnosis, 46 patients were excluded. The remaining 244 participants included 111 patients suffering from bladder cancer and 133 control patients visiting for other reasons than bladder cancer or urologic disease. The initial diagnosis of bladder cancer was based on cystoscopy and confirmed by histological and immunohistochemical examination of resected tissue. Tumors have been assigned as low- or high-grade tumors according to the 2004 WHO classification [58]. Urine was collected before transurethral resection. Tumor patients and controls were matched for age and gender. The study was approved by the ethics committee of the Landesärztekammer Brandenburg No. AS 147(bB)/2013 (17 November 2013).

### 4.2. Urine Sample Collection

Midstream urine samples were collected at the Urology department of the Lukaskrankenhaus Neuss, Germany. Urinary samples were processed immediately or stored at +4 °C for a maximum of 4 h. Urine status was assayed by routine dipstick analysis. For the UBC^®^ *Rapid* assay, three drops of fresh urine were used prior to further processing of a urine sample. After taking a 2-mL aliquot, remaining urine was centrifuged at 1500× *g* for 10 min at +10 °C in a swinging bucket rotor. Resulting supernatant was aliquoted (4 × 2 mL) and the pellet was resuspended in 1 mL of PBS. All samples were stored at −20 °C until further analyses were carried out. For further analysis, the samples were shipped on dry ice to the IPA in Bochum. 

### 4.3. UBC^®^ Rapid Test

The UBC^®^ *Rapid* test (Concile GmbH, Freiburg/Breisgau, Germany) was performed according to the manufacturers’ instructions as previously described [46]. The test cartridges were read out by the photometric point-of-care (POC) system Concile^®^ Ω100 reader (Concile GmbH) according to the manufacturers’ instructions, allowing a quantitative analysis of the test results.

### 4.4. Expression and Purification of Recombinant Survivin

Template cDNA of survivin (BIRC5) was obtained from Sino Biologicals, Beijing, China. The standard PCR protocol included the primers p1 (5′-GTATACCATATGGGTGCCCGG-3′) and p2 (5′-CGGATCCTCAATCCATGGCAGC-3′). The PCR product was cut with NdeI and BamHI (New England Biolabs, Ipswich, MA, USA) for an in-frame ligation of the survivin coding sequence into pET16b (Merck Millipore Novagen, Darmstadt, Germany), which provides the coding sequence for an N-terminal polyhistidine-tag. Cloning products were confirmed by sequencing. The resulting pET16b_His-Survivin plasmid was transformed into *E. coli* BL 21 CodonPlus RIPL (Agilent Technologies, Santa Clara, CA, USA) cells for heterologous protein expression. The cells of a 1 L culture were harvested and lysed by urea denaturation in 20 mM Hepes, 100 mM NaCl (pH 8). Purification of the His_10_-Survivin fusion protein was performed as described elsewhere [59]. Briefly, cell lysate was centrifuged to remove cell debris. Soluble supernatant was applied to an affinity chromatography column (HisTrap HP, GE Healthcare Life Science, Freiburg, Germany). After washing, the purified protein was eluted with elution buffer (20 mM Hepes, 100 mM NaCl, 8 M Urea, 250 mM imidazole, pH 8). Eluted affinity-purified His_10_-Survivin showed high purity and was further processed on a size-exclusion chromatography column (SuperdexTM 75, Amersham Bioscience, Little Chalfont, Buckinghamshire, UK) for protein refolding. Final proteins in PBS was stored at −20 °C and used for immunization of rabbits for the generation of polyclonal antibodies. 

### 4.5. Immunization and Antibody Purification

Immunization with His_10_-Survivin was performed at Charles River (Charles River Laboratories, Chatillon-sur-Chalaronne, France) following standard immunization protocols. After 70 days, 90 mL final sera were received and 20 mL of these were further processed for antibody purification. For this purpose, sera were loaded onto a Profinity Protein A column (Bio-Rad, Munich, Germany) linked to a Next Generation Chromatography System (Bio-Rad). The column was washed with PBS and the purified antibodies were eluted with 100 mM glycine (pH 2.8). Purified anti-survivin antibodies were immediately dialyzed against PBS yielding a final IgG concentration of 2.87 mg/mL (15 mL) as determined by absorption at 280 nm.

### 4.6. Biotinylation of Anti-Survivin Antibody

For ELISA development, the purified survivin antibody was partly biotinylated. Briefly, purified antibody was mixed with 33× molar excess of NHS-Biotin (Thermo Fisher, Waltham, MA, USA) in 10 mM NaHCO_3_ (pH 8.4), incubated for 3 h at room temperature and finally dialyzed against PBS. The final concentration of biotinylated antibody was 2.18 mg/mL (5 mL).

### 4.7. Survivin ELISA

In the experiments, 250 µL urinary cell pellet was centrifuged with 1500× *g* for 5 min at +4 °C to remove the storage solution. Afterwards, the pellet was completely dissolved in 500 µL CytoBuster (Merck Millipore, Darmstadt, Germany) and concentrated by using Vivaspin 500 spin columns (Sartorius, Stonehouse, Gloucestershire, UK) to 50 µL. To this solution, 150 µL of PBST (PBS with 0.05% Tween-20) was added and used as sample for measurement. Residual CytoBuster was tested to have no influence on antibody binding. Microtiter plates (Thermo Scientific, Roskilde, Denmark) were coated with purified polyclonal antibodies (100 μL/well, diluted 1:5000 in 100 mM carbonate/bicarbonate buffer, pH 9.6) and allowed to adhere overnight at +4 °C. They were then blocked with 1.5% casein, washed, and incubated with dilutions of the standard and samples for 1 h at room temperature. A stock solution of survivin was diluted in PBST to give standard concentrations between 5 and 0.02 ng/mL. Next, plates were washed three times with PBST and incubated for 1 h with biotinylated polyclonal antibodies (100 μL/well, dilution 1:10,000). After washing, 100 μL/well of 1:20,000 diluted horseradish-peroxidase-streptavidin conjugate (Fitzgerald Industries International, Concord, MA, USA) was added. After 1 h, plates were washed with PBST and 100 μL H_2_O_2_-activated ABTS substrate solution was added to each well. The enzyme reaction was stopped by addition of 100 μL 0.32% NaF and absorbance was read at 414 nm.

The dose–response curves for standards were obtained by 4-parameter curve fitting using SoftMax Pro 4.7.1 from Molecular Devices (Sunnyvale, CA, USA). The lower detection limit of the assay was defined by adding 0.05 OD units (2-fold mean of the background standard deviation of 20 plates) to the background value of each plate. Samples were considered as positive for survivin if the measured concentration was above the cut-off. The cut-off was defined as the limit of detection. Reproducibility experiments were performed with samples containing low (0.168 ng/mL), medium (0.667 ng/mL) and high (2.240 ng/mL) survivin concentrations.

### 4.8. Statistics

Statistical analyses were performed with SAS/STAT and SAS/IML software version 9.4 (SAS Institute Inc., Cary, NC, USA) or Prism 5 (GraphPad Software, Inc., San Diego, CA, USA). Plots were generated with Prism 5. Median and inter-quartile ranges (IQR) were used to describe the distribution of continuous variables. Groups were compared using the non-parametric Wilcoxon signed-rank test. Spearman’s correlation coefficients (r_s_) and 95% confidence intervals were used to describe rank correlations between variables. The performance of the individual biomarkers and their combination was evaluated by receiver operating characteristic (ROC) analysis. For the panel, an ‘or’ combination of both markers was used. ROC curves were compared with a non-parametric approach [60].

## 5. Conclusions

In summary, a new ELISA to quantitate survivin in urine samples has been developed and evaluated alongside the UBC^®^ *Rapid* test in a case-control study. Both assays detected bladder cancer, preferably high-grade tumors, but each with relatively low sensitivity. For the first time, survivin and UBC^®^ *Rapid* have been tested as a panel, demonstrating an increase in sensitivity. However, the results require further testing of the assays with other control groups, like patients at suspicion for bladder cancer, and finally validation in a prospective study using a high-risk cohort.

## Figures and Tables

**Table 1:** Characteristics of the study population and patients.

Characteristics	All (*n* = 244)			Tumor (*n* = 111)			Clinical Controls (*n* = 133)		
**Gender**	Male		Female	Male		Female	Male		Female
***n***	175		69	83		28	92		41
	***n***	**Median**	**IQR**	***n***	**Median**	**IQR**	***n***	**Median**	**IQR**
**Age (years)**	244	73	63–80	111	74	65–80	133	71	60–79
**Body mass index**	244	26.5	24.0–29.8	111	26.9	24.3–30.4	133	26.3	23.6–29.6
**Urine volume (mL)**	244	30	16–40	111	20	14–40	133	35	20–50
**Urine retention within bladder (h)**	236	2.0	1.0–3.0	106	1.75	1.0–2.5	130	2.0	1.0–3.5
**Specific gravity (g/L)**	240	240	1015	109	1015	1015–1020	131	1020	1015–1020
**pH**	240	5	5.0–6.5	109	5	5–6.5	131	5	5–6.5
**Survivin (ng/mL)**				111	0.014 *	0–0.528	133	0	0–0.249
**UBC^®^*Rapid* (mg/L)**				111	16.3	5–300	133	5	5–28.2

IQR = interquartile range; * extrapolated value.

**Table 2:** Characteristics of the tumor group.

Characteristic	Status	(*n*)
**Tumor Stage**	Ta	61
	T1	14
	T2	23
	T3	9
	Carcinoma in situ	3
	Missing	1
**Histological grade**	Low	55
	High	55
	Missing	1
**Recurrent**	Yes	52
	No	58
	Missing	1
**Gross hematuria**	Yes	66
	No	45
**Dysuria**	Yes	33
	No	78
**Frequent urination**	Yes	51
	No	60
**Bladder Stones**	Yes	12
	No	99
**Diabetes mellitus type II**	Yes	12
	No	99
**Smoking**	Never	23
	Former	51
	Actual	35
	Missing	2

**Table 3:** Performance of survivin, UBC^®^ *Rapid*, and combination based on single-marker cut-off for the detection of bladder cancer.

Groups	Cut-Off	Sensitivity (%)	Specificity (%)	True-Positive (*n*)	True-Negative (*n*)	False-Positive (*n*)	False-Negative (*n*)
**Survivin ELISA**							
Tumor vs. Controls	0.033 ng/mL	35	98	39	131	2	72
High-grade tumor vs. Controls		51	98	28	131	2	27
Low-grade tumor vs. Controls		18	98	10	131	2	45
**UBC^®^*Rapid***							
Tumor vs. Controls	10.0 mg/L	56	96	62	128	5	49
High-grade tumor vs. Controls		73	96	40	128	5	15
Low-grade tumor vs. Controls		40	96	22	128	5	33
**Combination**							
Tumor vs. Controls	>Survivin or >UBC^®^ *Rapid*	66	95	73	127	6	38
High-grade tumor vs. Controls		82	95	45	127	6	10
Low-grade tumor vs. Controls		49	95	27	127	6	28

## Supplementary Materials

Supplementary materials can be found at www.mdpi.com/1422-0067/19/1/226/s1.

## Abbreviations

AUC	Area under the curve
CV	Coefficient of variation
LoD	Limit of detection
ELISA	Enzyme-linked immunosorbent assay
IQR	Interquartile range
ROC	Receiver operating characteristic
