# Supplementary material for: Evaluation of a New Survivin ELISA and UBC® Rapid for the Detection of Bladder Cancer in Urine
Source: Int J Mol Sci. 2018 Jan 11;19(1):226. doi: 10.3390/ijms19010226 (PMC5796175; doi:10.3390/ijms19010226)
Supplement: Supplementary file 1 [file ijms-19-00226-s001.pdf]

International Journal of *Molecular Sciences*

MDPI

Article

Evaluation of a new survivin ELISA and UBC® Rapid for the detection of bladder cancer in urine

Jan Gleichenhagen[1*], Christian Arndt[2], Swaantje Casjens[1], Carmen Meinig[1], Holger Gerullis[3], Irina Raiko[1], Thomas Brüning[1], Thorsten Ecke[4] and Georg Johnen[1]

Supplementary Materials

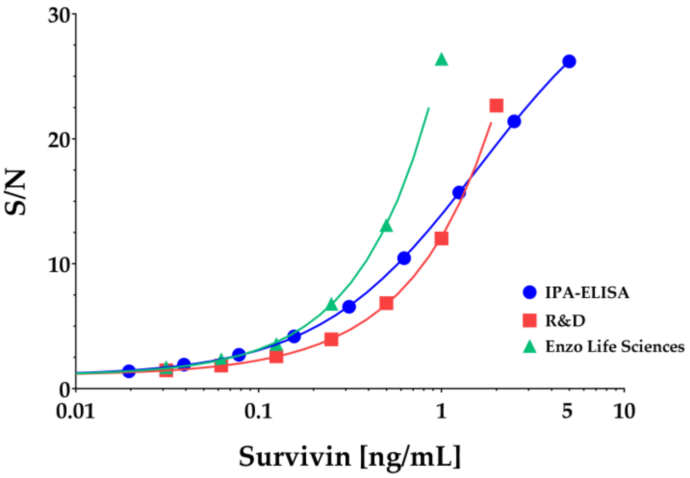

Supplementary Figure S1. Comparison of different survivin assays. Obtained OD values from two commercial assays (R&D, ENZO) and the new ELISA (IPA) are plotted as signal to noise ratio (S/N) and interpolated by four-parameter logistic curve fitting.

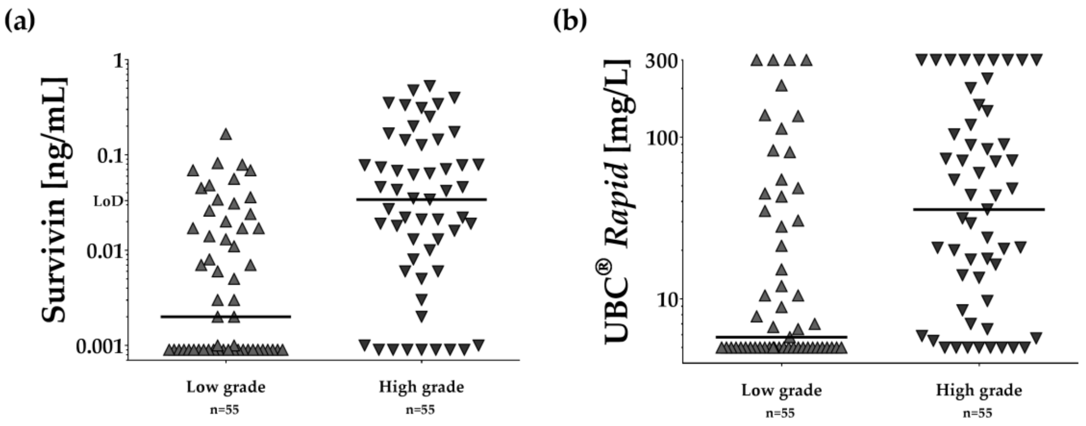

Supplementary Figure S2. Comparison of values for low-grade and high-grade tumors. (**a**) Survivin and (**b**) UBC® *Rapid*.

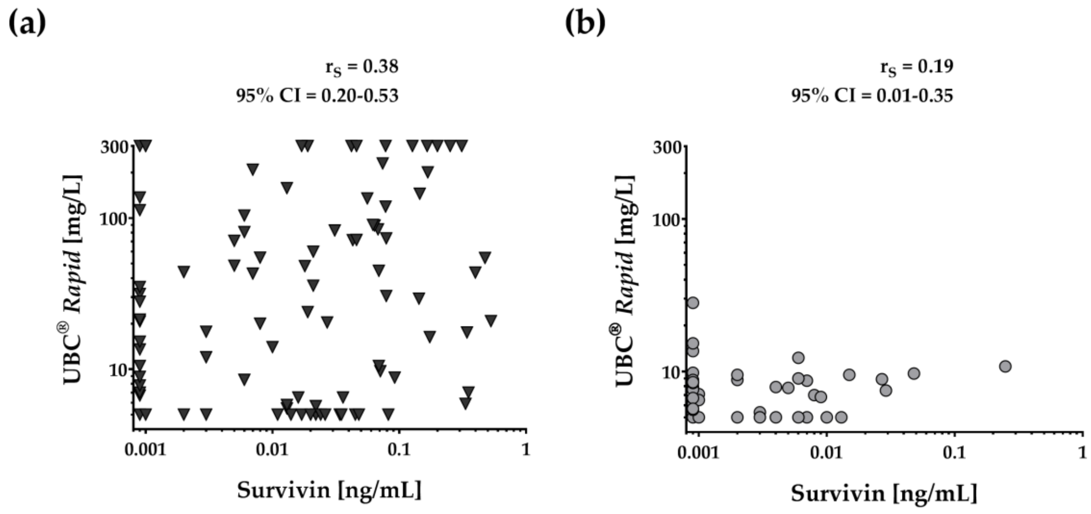

Supplementary Figure S3. Scatterplot of UBC® *Rapid versus* survivin. The plot shows marker concentrations of the (**a**) tumor group (r_s = 0.38) and (**b**) clinical control group (r_s = 0.19). CI = 95% confidence interval

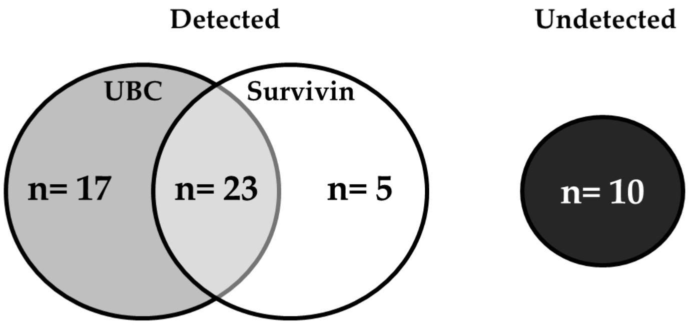

Supplementary Figure S4. Venn diagram of high-grade tumors. Detected (left) and undetected (right) tumor cases by UBC® *Rapid* (cut-off 10 mg/l) and survivin (cut-off 0.033 ng/ml).

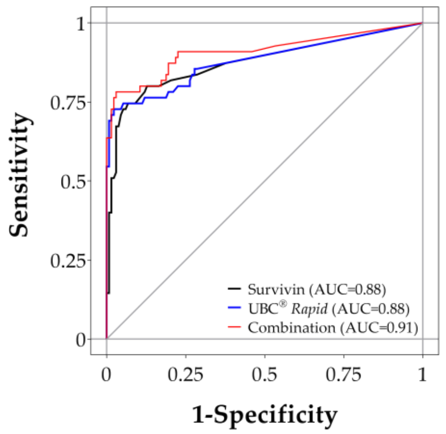

Supplementary Figure S5. ROC analyses of high-grade tumors and clinical controls for survivin (AUC = 0.88), UBC® *Rapid* (AUC = 0.88), and the combination (AUC = 0.91).
